# Evaluation of an Internet-Based Hearing Test—Comparison with Established Methods for Detection of Hearing Loss

**DOI:** 10.2196/jmir.1065

**Published:** 2008-10-21

**Authors:** Christin Bexelius, Louise Honeth, Alexandra Ekman, Mikael Eriksson, Sven Sandin, Dan Bagger-Sjöbäck, Jan-Eric Litton

**Affiliations:** ^2^Department of EarNose and ThroatKarolinska HospitalSolnaSweden; ^1^Department of Medical Epidemiology and BiostatisticsKarolinska InstitutetStockholmSweden

**Keywords:** Hearing tests, audiometry, pure-tone, Internet, questionnaires, epidemiology, cohort study

## Abstract

**Background:**

Hearing impairment is most accurately measured by a clinical pure-tone audiogram. This method is not suitable for large-scale, population-based epidemiological studies as it requires that study participants visit a clinic with trained personnel. An alternative approach to measuring hearing ability is self-estimation through questionnaires, but the correlation to clinical audiometric tests varies.

**Objective:**

To evaluate an Internet-based hearing test pilot compared to a question about self-estimated hearing and the feasibility of using an Internet-based hearing test and an Internet-based questionnaire in a population of 560 members of the Swedish Hunters’ Association in the age group 20-60 years.

**Methods:**

An invitation was mailed to the participants in March 2007 together with the URL to the study Web site, a personal username, and a password. The Web site included the questionnaire, the hearing test, and instructions for participating in the study. The hearing test resembles a clinical audiogram presenting 6 tones between 500 and 8000 Hz. Tones are presented between 0 and 60 dB, and the participant responds to the tones by pressing the space bar. The hearing test requires headphones and is based on JAVA programming. Before the participant can start the hearing test, it has to be calibrated against a reference person with good hearing between 15 and 35 years of age.

**Results:**

After 5 months, 162 out of 560 (29%) had answered the questionnaire, out of which 88 (16%) had completed the hearing test. Those who actively declined participation numbered 230 out of 560 (41%). After removing duplicates and hearing tests calibrated by unreliable reference data, 61 hearing tests remained for analysis. The prevalence of hearing impairment from the Internet-based hearing test was 20% (12 out of 61), compared to 52% (32 out of 61) from the self-estimated question. Those who completed the hearing test were older than the non-participants, and more had headphones (*P* = .003) and the correct version of the JAVA program (*P* = .007) than those who only answered the questionnaire.

**Conclusions:**

Though an Internet-based hearing test cannot replace a clinical pure-tone audiogram conducted by a trained audiologist, it is a valid and useful screening tool for hearing ability in a large population carried out at a low cost.

## Introduction

Hearing loss is one of the most common physical impairments in the western world and is an increasing problem among younger age groups [1]. The gold standard for estimating hearing impairment is a clinical pure-tone audiogram [2]. This method is not suitable for large-scale, population-based epidemiological studies as it demands access to equipment and trained personnel and is demanding for the participants in terms of travel to a clinic. An alternative approach for estimating hearing in epidemiological studies is self-estimation from a set of questions [3,4]. However, the sensitivity of these self-estimated hearing approaches varies, and their correlation to pure-tone audiograms is arguable [4-10]. Though self-estimated hearing approaches might be efficient in measuring a patient’s reactions and the social impact of hearing loss [11], they cannot replace audiometric hearing tests [4].

Digital technologies provide the possibility of developing computer-based programs for measurement of physical impairment. A number of commercial programs resembling clinical audiograms for measuring hearing are available online [12,13]. Various Internet-based and computerized audiology systems for measuring hearing thresholds of patients have been developed and tested in different studies [14-17]. The systems are developed to evaluate patients with a suspected hearing impairment at remote sites where access to trained audiologists and clinical pure-tone audiograms is limited. These systems are connected to a conventional audiometer and controlled via the Internet, or they require specific sound cards and modules. Trained personnel are still required, and the systems cannot be used for self-screening of hearing in large-scale epidemiological studies.

We have developed an Internet-based hearing test resembling a clinical pure-tone audiogram. The hearing test aims at measuring real-time hearing ability in large-scale epidemiological and clinical studies in the participant’s home environment, using headphones and a home computer. The Internet-based hearing test has been validated against a pure-tone audiogram at the Karolinska University Hospital [18]. Out of 72 individuals, 20 individuals were diagnosed with a moderate or severe hearing loss (greater than 40 dB) according to the pure-tone audiogram.The Pearson’s correlation coefficient between the two tests was 0.94 (*P*-value < .001) for the right ear and 0.93 (*P*-value = .001) for the left, and the Internet-based hearing test had a 75% sensitivity and a specificity of 96% compared to the clinical audiogram. The Internet-based hearing test is not a substitute for pure-tone audiometry for diagnosis of hearing loss, but rather should be used to screen for hearing ability in longitudinal, large-scale, population-based studies. Therefore, the sensitivity is sufficient.

This paper evaluates the pilot study testing the feasibility of collecting epidemiological data on hearing ability using an Internet-based hearing test together with an extensive questionnaire including questions about self-estimated hearing prior to the test. The study is also evaluated in terms of willingness to participate and possible reasons for non-participation, including technical obstacles.

## Methods

### Study Method

A pilot study was designed to test the feasibility of conducting a large-scale cohort study among more than 200,000 hunters and marksmen from the Swedish population. The larger study aims at studying the relationship between noise-induced hearing loss, exposure to heavy gun shots, and the use of hearing protection.

The participants enter the study through a Web site that includes a Web-based questionnaire and an Internet-based hearing test. To enter the Web page, the participant enters a personal username and password. The participants cannot access the hearing test before filling in the questionnaire. The questionnaire includes 12 sections with, in total, approximately 100 questions regarding background, hunting, self-estimated hearing, occupation, military service, problems with hearing, medications, and recreational activities. The question about self-estimated hearing was stated as, “How is your hearing?”, and the optional answers were “good”, “minor hearing loss”, “moderate hearing loss”, or “severe hearing loss”.

The hearing test is based on JAVA 5.0, and before the participants can start the hearing test, they are instructed to verify whether or not the computer has the correct version of the JAVA program. If not, the JAVA program can be downloaded free of charge. Before the participants can start the hearing test, the sound levels are calibrated against a reference person to compensate for variations in different headphones and noise interference from the computer and surroundings. Prior to the calibration test, the participant and the reference person are instructed to follow guidelines on how to set correct volume settings on the computer as well as using the headphones. In the following calibration phase, the reference person enters age (preferably between 14 and 35 years) and gender. The reference person is presented with a volume slider having a fine-tuned scale ranging over 30 dB. The reference person is instructed to move the slide head to a barely audible position, which is the reference hearing level (RefHL) for the frequency, and then request the program to present the next tone. The tone is a frequency-modulated sinus tone—a slightly vibrating tone which can be heard on headphones having “dead points” at certain pure frequencies. This tone is presented to both ears to get the lowest hearing threshold for each ear. The procedure starts from 500 Hz and is repeated for 1000 Hz, 2000 Hz, 4000 Hz, 6000 Hz, and 8000 Hz.

Quality check of the calibration of the RefHL data is performed on the finalized data. It is limited to a maximum check of a 15 dB difference over the frequencies, along with a check of whether the reference person has moved the volume slider for each frequency.

During the hearing test, intensity levels are presented between 0 (from reference calibration) and 60 dB sound pressure level (dBSPL). The hearing test starts by presenting the 500 Hz tone to the left ear for 1 second at 30 dBRefHL, which is 30 dB higher than the hearing threshold set by the reference person for that frequency. The tone is followed by a shorter pause of random length to discourage the participant from guessing. The participant presses the space bar on the computer keyboard to register that a tone is heard. The key press is accepted as registering a threshold if the key is pressed within the presented tone timeframe, adjusted for the human reaction time. If the space bar is pressed half a second after the accepted timeframe, it is registered as too late and considered “imagined”. This is not accepted as a threshold measure. When a tone is registered as heard, the test presents the same frequency at a 6 dB lower intensity level. When a tone is not heard, the test instead presents a tone at a 6 dB higher intensity level. The test proceeds for both left and right ears to settle the hearing levels for each frequency. This test procedure is a Web adaptation of established clinical audiometric testing and follows the guidelines for clinical audiometric testing [19].

After completing the test, the participant is shown an audiogram presenting the hearing levels for both ears at each measured frequency.

### Recruitment

In March 2007, an invitation letter was sent to 560 members of the Swedish Hunters’ Association. Subjects were selected proportionally to the distribution among the members in terms of gender (men = 500, women = 60) and age (in the age group 20-60). The mailed invitation included a description of the study and a personal username and password. The invitation also included a prepaid return letter which the participants could use to decline participation. This letter included a voluntary question about their reason for non-participation. The data collection was closed in August 2007. During the study, 2 paper reminders were sent, followed by a telephone reminder. The first reminder was sent 3 weeks after the initial invitation, followed by a second reminder after an additional 3 weeks and a telephone reminder 3 weeks after the second paper reminder. During the telephone reminder, those who declined participation were asked about the reason for their non-participation. Reminders were sent to subjects who had not yet completed the questionnaire and hearing test without declining participation, and to those who had answered the questionnaire but not completed the hearing test.

### Statistical Analysis

The audiometric data from the hearing test was classified according to the definition by WHO for normal hearing, minor hearing loss, moderate hearing loss, and severe hearing loss [20]. Normal hearing was set as between 0 and 25 dB on all frequencies. The cut-off level for minor hearing loss was 1 or more frequency-values in the range 26-40 dB; for moderate hearing loss 1 or more frequency-values in the range 41-60 dB; and for severe hearing loss 1 or more frequency-values higher than 60 dB on either frequency. The result of the hearing test was compared to the self-estimated hearing question from the questionnaire prior to the hearing test and graded on the above scale (no hearing loss to severe hearing loss). The analysis is based on the audiometric data from the best ear in the hearing test. The 2 hearing tests (Internet-based hearing test and self-estimating question) were compared by using a contingency table presenting individuals categorized with normal hearing, minor hearing loss, moderate hearing loss, and severe hearing loss.

 The study procedure is described with respect to the compliance and dropout at different checkpoints throughout the study (Table 1). Background data on age and gender were provided from the Swedish Hunters’ Association for comparison of non-respondents, participants who declined, and respondents. Full respondents were compared with participants who had answered the questionnaire but had not completed the hearing test (questionnaire respondents) on the basis of different background variables including age, gender, level of education, and number of individuals in household. To evaluate the different technical steps, the full respondents were also compared with the questionnaire respondents as to whether or not they had headphones in their home prior to the test, if they had the correct version of JAVA installed on their computers, and their willingness to provide their e-mail addresses for future contact. The Pearson Chi-Square test was calculated to test if the distribution of subjects across demographic variables between full compliers and questionnaire completers was equal (Table 2). All tests of the statistical hypothesis were made on the two-sided 5% level of significance. To calculate the agreement between the hearing test and the self-estimated hearing, a simple kappa coefficient was used where agreement was corrected for chance [21]. All presentations and data evaluations were made utilizing the SAS 9.1.3 software. The regional ethical committee approved the study in October 2006.

## Results

### Response Rate

**Figure 1:**
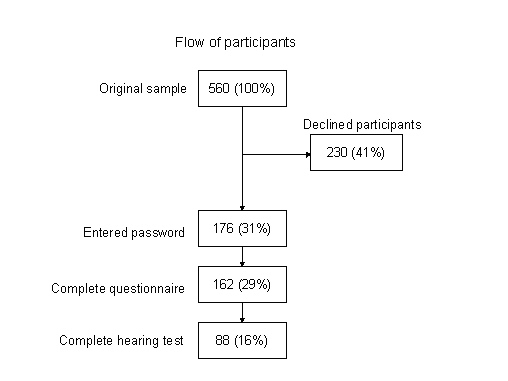
Flowchart of participation in the pilot study for evaluating an Internet-based hearing test among 560 members of the Swedish Hunters’ Association

After 3 reminders, 162 out of 560 (29%) had completed the questionnaire (questionnaire respondents), of which 88 (16%) had completed the hearing test (full respondents). After reminders 1 and 2, 146 had actively declined participation, and an additional 84 declined participation during the telephone reminder (total 230, 41% of the total sample). There were 154 individuals who could not be reached or did not contact the study center for non-participation, and 14 participants entered the password without completing the study. A flowchart of the participation scheme is presented in Figure 1.

### Hearing Test

In total, 126 hearing tests were carried out by 88 unique participants. Among the duplicates, the test with the best (eg, smallest degree of hearing loss) result was used in the analysis. After removal of those hearing tests with an incorrect reference, 61 hearing tests remained for which the mean age was 45 years. Results of the hearing test in comparison to the self-estimated hearing question are shown in Table 1.

On the self-estimated hearing question, 32 out of 61 (52%) reported hearing loss; 12 of those 61 (20%) showed hearing loss on the Internet-based hearing test. The Chi-Square test shows this difference to be statistically significant (*P* < .001). Only one who had a higher degree of hearing loss had a documented ear injury. Among those who had a hearing impairment according to the Internet-based hearing test, 6 out of 12 (50%) had classified their hearing differently in the self-estimated question. After excluding severe hearing loss, the simple kappa coefficient was calculated to 0.18 (95% confidence interval 0.005-0.359), indicating a slight agreement between the two measurements.

**Table 1:** Correlation of hearing test to the self-estimated hearing question (61 individuals)

		Self-estimated hearing loss, 1 question				
		No	Minor	Moderate	Severe	
Hearing test							
	No	27 (55%) (93%)	21 (43%) (84%)	1 (2%) (14%)	-	49 (80%)
	Minor	2 (29%) (7%)	3 (43%) (12%)	2 (29%) (29%)	-	7 (11%)
	Moderate	-	1 (25%) (4%)	3 (75%) (43%)	-	4 (7%)
	Severe	-	-	1 (100%) (14%)	-	1 (2%)
		29 (48%)	25 (41%)	7 (11%)	-	61

### Sociodemographic Distribution

The distribution of gender was similar in all groups of respondents, the original sample, declined participants, and non-respondents (Table 2). The full respondents were older than the non-respondents and questionnaire respondents. Statistically, the full respondents were not significantly different from the questionnaire respondents in terms of sociodemographic characteristics or self-estimated hearing prior to the test (Table 3). When looking at the technical attributes, full respondents were more likely to have headphones at home (*P* = .003) and the correct JAVA version on their computers (*P* = .007) compared to questionnaire respondents (Table 2).

Common reasons for declining participation were lack of time (17%), lack of interest in the study (35%), lack of headphones (13%) (which reflects a difference between questionnaire respondents and full respondents), having no experience of gunshots or hunting, or already experiencing hearing loss and therefore considering themselves to be inappropriate for the study (Table 4).

**Table 2:** Distribution of age and gender among all participants in the pilot study for evaluating an Internet-based hearing test among 560 members of the Swedish Hunters’ Association

	Non- respondents n = 154 (28%)	Declined study n = 230 (41%)	Drop outs/ Lurkers n = 14 (3%)	Answered questionnaire only (Questionnaire respondents) n = 74 (13%)	Hearing test and questionnaire (Full Respondents) n = 88 (16%)	Total n = 560 (100%)
**Gender**						
Men	138 (90%)	200 (87%)	13 (93%)	68 (92%)	81 (92%)	500 (89%)
Women	16 (10%)	30 (13%)	1 (7%)	6 (8%)	7 (8%)	60 (11%)
**Age category**						
20-34	51 (33%)	57 (25%)	3 (21%)	17 (23%)	17 (19%)	145 (26%)
35-49	63 (41%)	110 (48%)	7 (50%)	35 (47%)	39 (44%)	254 (45%)
50-60	40 (26%)	63 (27%)	4 (29%)	22 (30%)	32 (36%)	161 (29%)

**Table 3:** Sociodemographic characteristics among questionnaire respondents and full respondents for evaluating an Internet-based hearing test among questionnaire and full responders

	Answered questionnaire only (Questionnaire respondents) n = 74 (%)	Hearing test and questionnaire (Full respondents) n = 88 (%)	Pearson’s Chi-Square	*P*-value
**Gender**				
Men	68 (92%)	81 (92%)	0.001	0.97
Women	6 (8%)	7 (8%)		
**Age category**				
20-34	17 (23%)	17 (19%)	0.86	0.65
35-49	35 (47%)	39 (44%)		
50-60	22 (30%)	32 (36%)		
**Household Members**				
1	12 (16%)	6 (7%)	5.20	0.16
2	26 (35%)	27 (31%)		
3-4	31 (42%)	43 (54%)		
5-6	5 (7%)	11 (69%)		
Missing		1 (1%)		
**Education**				
Preschool	9 (12%)	9 (10%)	1.03	0.80
High School	30 (41%)	34 (39%)		
College/ University	35 (47%)	43 (49%)		
Missing		2 (2%)		
**Environment**				
Large city	10 (13%)	8 (10%)	2.50	0.64
Suburb	11 (15%)	9 (10%)		
Medium-sized city	10 (14%)	14 (16%)		
Small town	14 (19%)	23 (26%)		
Countryside	29 (39%)	33 (38%)		
Missing		1 (1%)		
**Java**				
Yes	23 (31%)	44 (50%)	7.31	0.007
No	51 (69%)	40 (45%)		
Missing		4 (5%)		
**Reported email**				
Yes	73 (99%)	86 (98%)	0.19	0.66
No	1 (1%)	2 (3%)		
**Have headphones at home prior to test**				
Yes	48 (39%)	74 (60%)	8.88	0.003
No	26 (61%)	13 (30%)		
Missing		1 (1%)		
**Self-estimated hearing prior test**				
No loss	44 (59%)	39 (44%)	7.35	0.06
Minor loss	18 (24%)	35 (40%)		
Moderate loss	8 (24%)	12 (14%)		
Severe loss	4 (5%)	1 (1%)		
Missing		1 (1%)		

**Table 4:** Reasons for declining participation in the pilot study for evaluating an Internet-based hearing test among 560 members of the Swedish Hunters’ Association

Non-participation Reason	After paper reminders 1 and 2 n = 146	After telephone reminder n = 84	Total n = 230
Have hearing loss prior study	5 (3%)	1 (1%)	6 (3%)
Have no computer	7 (5%)	10 (12%)	17 (7%)
Have no headphones	27 (18%)	4 (5%)	31 (13%)
Have no reference	3 (2%)	1 (1%)	4 (2%)
Don’t trust technique	3 (2%)	-	3 (1%)
Not interested	62 (42%)	19 (23%)	81 (35%)
Have no time	13 (9%)	26 (31%)	39 (17%)
No experience of hunting	17 (12%)	15 (18%)	32 (14%)
Computer problem	5 (3%)	8 (10%)	13 (7%)
Other	4 (3%)	-	4 (2%)

## Discussion

This study evaluates an Internet-based hearing test in terms of its agreement to self-estimated hearing assessed by a question in a questionnaire and willingness to participate. Statistically, the results from the hearing test and the self-estimated hearing were significantly different (*P* < .001). The Internet-based hearing test indicated hearing loss in 20% of the tested individuals, compared to 52% in the self-estimated question. These results could indicate an underestimation of self-estimated hearing ability and display the difficulty of evaluating a self-estimated hearing impairment. The high degree of underestimation could be a result of the difficulties in the calibration procedure of the Internet-based hearing test, resulting in minor hearing loss not being detected. But, as this study population is relatively young (20-60 years), 52% seems to be a high prevalence of hearing impairment, even though the study includes a population with high exposure to impulse noise. In 2005, 14.3% of the Swedish population had a hearing impairment, out of which 63% were still of working age (16-64 years) [22]. This figure is more comparable to the Internet-based hearing test than to the self-estimated hearing. Many of the validated questionnaires and questions measuring self-estimated hearing ability have been evaluated on older populations with a high prevalence of hearing loss [2,4,5,8,9,10] and are, therefore, difficult to use on a younger population with a low prevalence of hearing loss. Self-estimated questionnaires cannot measure noise-induced hearing loss in terms of changes in frequency-specific impairments and can, therefore, not replace a clinical audiogram [4]. This strengthens the need for a more objective tool for measuring hearing ability in larger samples. The high prevalence of self-estimated hearing loss among the full respondents could, however, be biased by the fact that it was answered predominantly by people with hearing loss while people with no hearing loss refrained from participating.

The study also aims at evaluating the willingness to take part in a study including a Web-based questionnaire and an Internet-based hearing test. Our study had a response rate of 29% to the questionnaire and 16% to the hearing test, which is low for an epidemiological study. Full respondents were slightly older than the average non-participant, which might indicate that the older age group had a keener interest in the study. This was expected, as hearing decreases with age.

There were no differences between questionnaire respondents and full respondents in terms of sociodemographic characteristics and self-estimated hearing, where the full respondents were a representative sample of the total respondents. The full respondents had, however, greater access to headphones and already possessed the correct version of JAVA prior to the test more often than did the questionnaire respondents. The low response rate might therefore be due to the technique and the many steps prior to the test (including the need for acquiring headphones, JAVA, and a reference person), rather than personal characteristics. One concern prior to the study was computer and Internet knowledge among the study participants, but Internet use in Sweden is among the highest in the world. In Sweden, 96% of the population can access the Internet from their homes [23], and an increasing number of households have broadband with a high-speed connection [24]. Therefore, the Swedish population is a suitable target group for this kind of study. When looking at non-respondents, the primary reason for non-participation is probably lack of interest. Of the non-respondents, 14% said that they had no experience of firing during hunting and therefore felt they were not the correct target group for the study. According to the Swedish Hunters’ Association, 5% of the members do not hunt, but many of the members are involved in hunting without firing. In the invitation letter, the relationship between heavy gun shots and hearing impairment during hunting was mentioned, thus this group of non-participants might have misunderstood the invitation. To raise the response rate in the large-scale study, the information in the invitation letter should be enhanced and possibilities for subvention of headphones should be investigated. The large-scale study aims at recruiting 50,000 individuals.

One of the major problems of this study was the calibration and especially the quality of the reference data, as many of the respondents seemed to use a reference person with unreliable hearing. The ability to test the hearing of the reference person is limited. Other Internet-based hearing tests have used a reference tone or a specific program for calibrating the zero level [12,15]. This is problematic, however, as noise levels of computers and the surrounding environments, as well as the quality of headphones, vary for each individual and setting. In a small pre-study, different headphones were evaluated in terms of sound-levels on different frequencies, and differences between the different headphones and frequencies were found. Many of the headphones had “dead points” where the tone had reduced intensity or was distorted at specific frequencies. This problem was reduced by using a frequency modulated sinus tone that is a slightly vibrating tone instead of a pure sinus tone. The individuals in the study were instructed to calibrate the system and perform the test in an environment as silent as possible. This is no guarantee for excluding environmental noise. However, the validation study performed parallel to this study showed surprisingly small differences between the Internet-based test and the pure-tone audiogram in the lower frequencies (500 Hz and 1000 Hz), indicating that these frequencies were not badly affected by environmental noise. Nor were other frequencies effected, and the highest mean difference between the two tests was 5 dB [18].

For the large-scale study, the calibration technique will be redesigned to measure the reference threshold twice in order to get more reliable values and to better judge that a reference is suitable (hearing loss estimated to be less than 15 dB). Also, the reference person will be asked to answer a couple of questions regarding hunting experience and perceived hearing in order to detect potential bias.

Hearing impairment is a growing problem and can occur at all ages. Causes include repetitive exposure to loud sounds, or other external noises [25]. Hearing loss is a social handicap and can often lead to a decrease in quality of life and premature retirement [3,26]. As hearing ability decreases naturally with age, a minor hearing loss caused by noise at a younger age can become a greater problem later in life [27,28]. A major challenge in treating hearing loss is early identification. If hearing ability is decreased in one ear at a young age, it is often compensated for by the better ear. When hearing ability is decreased naturally with age, the acquired hearing loss increases the problem. Prospective longitudinal epidemiological studies can increase the knowledge about the development of hearing loss and preventive measures. The Internet-based hearing test in this study has been validated against a clinical pure-tone audiogram and provides the benefit of an objective and cost effective alternative to screening hearing ability on 6 different frequencies. It can also detect changes in hearing impairment over time when used in longitudinal epidemiological studies.

Though the Internet-based hearing test cannot replace an audiogram from a clinical pure-tone audiometer conducted by a trained audiologist, it is a more useful and objective tool for screening hearing in a large population than a self-estimated hearing questionnaire.

## Abbreviations

JAVA: Java technology was created as a computer programming tool at Sun Microsystems in 1991
dB: decibel
dBRefHL: dB reference hearing level
dBSPL: dB sound pressure level
Hz: Hertz
URL: Uniform Resource Locator
WHO: World Health Organization
